# The Effectiveness of a Computer Game-Based Rehabilitation Platform for Children With Cerebral Palsy: Protocol for a Randomized Clinical Trial

**DOI:** 10.2196/resprot.6846

**Published:** 2017-05-18

**Authors:** Anuprita Kanitkar, Tony Szturm, Sanjay Parmar, Dorcas BC Gandhi, Gina Ruth Rempel, Gayle Restall, Monika Sharma, Amitesh Narayan, Jeyaraj Pandian, Nilashri Naik, Ravi R Savadatti, Mahesh Appasaheb Kamate

**Affiliations:** ^1^ Applied Health Sciences University of Manitoba Winnipeg, MB Canada; ^2^ College of Rehabilitation Sciences University of Manitoba Winnipeg, MB Canada; ^3^ SDM College of Medical Sciences and Hospital Rajiv Gandhi University of Health Sciences Dharwad India; ^4^ Christian Medical College and Hospital Department of Neurology Baba Farid University of Health Sciences Ludhiana India; ^5^ Max Rady College of Medicine, Rady Faculty of Health Sciences Department of Pediatrics and Child Health University of Manitoba Winnipeg, MB Canada; ^6^ Christian Medical College and Hospital Department of Pediatrics Baba Farid University of Health Sciences Ludhiana India; ^7^ Kasturba Medical College Department of Physiotherapy Manipal University Mangalore India; ^8^ Department of Physiotherapy Ushas School for Exceptional Children Hubli India; ^9^ SDM College of Physiotherapy Rajiv Gandhi University of Health Sciences Dharwad India; ^10^ JN Medical College and Hospital Department of Pediatrics KLE University Belgaum India

**Keywords:** repetitive task practice, cerebral palsy, fine motor skills, game-based exercise, randomized controlled trial, upper extremity function

## Abstract

**Background:**

It is difficult to engage young children with cerebral palsy (CP) in repetitive, tedious therapy. As such, there is a need for innovative approaches and tools to motivate these children. We developed the low-cost, computer game-based rehabilitation platform CGR that combines fine manipulation and gross movement exercises with attention and planning game activities appropriate for young children with CP.

**Objective:**

The objective of this study is to provide evidence of the therapeutic value of CGR to improve upper extremity (UE) motor function for children with CP.

**Methods:**

This randomized controlled, single-blind, clinical trial with an active control arm will be conducted at 4 sites. Children diagnosed with CP between the ages of 4 and 10 years old with moderate UE impairments and fine motor control abnormalities will be recruited.

**Results:**

We will test the difference between experimental and control groups using the Quality of Upper Extremity Skills Test (QUEST) and Peabody Developmental Motor Scales, Second Edition (PDMS-2) outcome measures. The parents of the children and the therapist experiences with the interventions and tools will be explored using semi-structured interviews using the qualitative description approach.

**Conclusions:**

This research protocol, if effective, will provide evidence for the therapeutic value and feasibility of CGR in the pediatric rehabilitation of UE function.

**Trial Registration:**

Clinicaltrials.gov NCT02728375; http:https://clinicaltrials.gov/ct2/show/NCT02728375 (Archived by WebCite at http://www.webcitation.org/6qDjvszvh)

## Introduction

### Background and Rationale

Canada and India face a growing population of children with cerebral palsy (CP), with the condition occurring in 2 to 4 of every 1000 live births in North America and India, respectively [1-3]. Children with CP have deficits in fine and gross motor skills, often with co-occurring deficits in visual-spatial processing skills [4-6]. The ability to perform functional tasks with the upper extremities (UEs) is an important predictor of success in daily activities and participation in school, leisure, and social activities [7]. Therapy programs designed to improve UE motor and visual-spatial processing skills must strive to maximize neurodevelopmental capacities and prevent secondary disabilities [8,9].

There are different approaches to therapy for children with CP [10-17]. The effectiveness of these programs is proportional to the intensity and amount of training and the task-specificity of the exercise regime [18-21]. For example, constraint-induced movement therapy (CIMT) and hand-arm bimanual intensive therapy (HABIT) are promising rehabilitation programs for restoration of hand-arm function. These treatment approaches stress that both functional demands and repetitive intensive training are important in the rehabilitation of fine motor skills and to restore functional skills. Typically CIMT needs administration of 6 hours a day and the child can use only the affected arm, making bimanual activities impossible to perform. There is growing evidence to support the idea of activity-dependent central nervous system (CNS) plasticity [22]. In addition, the notion is emerging that neural reorganization reflects learning achieved through generating real experiences, applying focused attention, simulating close-to-normal movements, and repetition [23]. However, it is difficult to engage children with CP in therapy for long periods of time and sustain motivation for the intense repetitive task practices. Thus, there is a need for innovative and cost-effective therapeutic approaches and tools that motivate children with CP to complete long-term neurorehabilitation programs and that provide opportunities to improve neurodevelopmental outcomes.

Parents and clinicians rate motivation as the most influential personal characteristic for adherence to therapy and for determining motor and functional outcomes in children with CP [24]. An emerging, promising approach to engaging children in therapy is to incorporate computer games in which a range of learning elements with interactive cognitive challenges help children to positively engage in activities. Studies have provided evidence of the benefits of video games in rehabilitation training and show that well-designed interactive games can improve players' motor skills and visual-spatial processing skills [24-30].

For this purpose, we developed the low-cost, computer-aided, game-based rehabilitation and learning platform CGR [31-37]. CGR combines fine or gross motor exercises and visual-spatial cognitive activities appropriate for children with CP in a game-based format. A motion detecting “Therapy Mouse” (Mobility Research, AZ) will be used as the computer game controller. It is a miniature and wireless plug-n-play computer interface device, which contains firmware and inertial sensors that allow physical motion, specifically instantaneous position, to be translated and interpreted as a standard motion of a Universal Serial Bus (USB) mouse, and which has high fidelity and responsiveness. Because the miniature motion mouse can be easily attached with Velcro to many objects, this approach provides a highly flexible therapy tool applied to fine or gross UE motor skills. Many objects with varied sizes, shapes, weights, surface properties, and functional demands can be used for exercise and for practicing a variety of gross or fine motor skills. Importantly, when the motion mouse is attached to the chosen object fun computer games can be played [8,28,31,36-37]. As CGR allows handling and manipulation of many objects (ie, ones commonly used in daily activities), activity goals can be imbedded in the therapy program. Many inexpensive modern games, “exergames,” and brain fitness games now exist that are visually rich, fun, and engaging, include a variety of visual-spatial tasks, and require choice and other planning type activities. Performing goal-directed manipulation tasks through engaging and guided repetition creates experiences crucial to improving the brain's ability to learn [10,37].

### Study Objectives

A randomized controlled trial (RCT) with an intention-to-treat is proposed to evaluate the effectiveness of the game-based rehabilitation program on fine manual dexterity, upper limb motor skills, and visual-spatial cognitive functions in children aged 4 to 10 years old diagnosed with CP. This single-blind randomized clinical trial with an active control arm will be conducted at 4 sites. Two groups of children will be examined: one group will receive the experimental game-based program and the other group will receive usual therapy (see Multimedia Appendix 1). The first hypothesis is that an engaging, game-based UE exercise regime will result in greater improvements in hand-arm function as compared to the usual outpatient physical therapy program. The second hypothesis is that the UE exercise program, which uses computer games having a variety of visual-spatial activities, will result in greater improvements in visuospatial cognitive functions as compared to the usual outpatient physical therapy program.

### Study Design

This study will evaluate the feasibility of the procedures such as recruitment, intervention delivery, participant retention, and measurement tools. Semi-structured interviews will be conducted with the parents of the children and with the treating therapists. The broad research questions are: “what were the experiences of the study participants with the game-based and current therapy programs, and on what context were the experiences based?” The qualitative findings of participant's and therapists' experiences will help to identify (1) perceived exercise benefits; (2) difficulties with the exercises and using the technologies; (3) engagement and motivational value of the computer games; (4) personal and environmental factors that influenced doing the exercises; and (5) recommendations and modifications for improving the exercise programs.

## Methods

### Study Setting

This randomized controlled, single-blind clinical trial with an active control arm will be conducted at the following 4 sites: (1) University of Manitoba and Rehabilitation Centre for Children at the Special Services for Children and Youth (SSCY) Centre (Drs Szturm, Rempel, Restall, and Mrs Kanitkar, Winnipeg, Manitoba, Canada); (2) SDM College of Physiotherapy, Dharwad in collaboration with Usha's School for Exceptional Children, Hubli (Drs Parmar, Savadatti, Kamate, and Naik, Karnataka, India); (3) Christian Medical College (Drs Sharma, Pandian, and Gandhi, Ludhiana, Punjab, India); and (4) Kasturba Medical College (Dr Narayan, Mangalore, Karnataka, India).

### Inclusion Criteria

Children diagnosed with CP (N=140) between the ages of 4 and 10 years old with moderate UE impairments and fine motor control abnormalities will be recruited. The following screening tools will be used: (1) Manual Ability Classification System (MACS), level 2 to 3 [38]; (2) Gross Motor Function Classification Scale (GMFCS), levels 2 to 4 [39]; (3) Ashworth scale of spasticity in wrist and fingers, level 0 to 1+ [40]; and (4) the pediatric version of the Mini-Mental State Examination assessment scale, level 17 and above. This will be used to the screen level of cognitive function. For each site, we will use a permuted block randomization scheme stratified by age and level of impairment as measured by the MACS and GMFCS [41].

### Exclusion Criteria

Exclusion criteria for the study will be (1) visual or auditory impairment such that they cannot see and interact with the video games; (2) secondary orthopedic complications due to neurodegenerative disease (NDD) or as a result of surgery to the upper limb that may have caused permanent changes in upper limb musculoskeletal structure; (3) recent Botulinum toxin therapy (less than 6 months); (4) seizures, or (5) complex communication disorders.

### Procedures and Interventions

Ethical approval was obtained from the health research ethics boards of each site. For each site a permuted block randomization scheme will be used and stratified by age where 4- to 6-year-olds will be one subgroup and 6- to10-year-olds will be the other subgroup. Each program will take 16 weeks, with 3 45-minute sessions per week. A workshop and uniform training program will be organized at SDM College of Physiotherapy, Dharwad, India for physiotherapists who will provide the assessment and the 2 intervention programs. This will be attended by Dr Szturm and Mrs Anuprita Kanitkar who will organize and coordinate the therapy program. The 2 intervention groups will be treated in all 4 locations limiting biases like contamination. The therapists conducting the assessments will be blinded to group assignment.

### Control Group Intervention

The control group (n=70) will receive the usual, comprehensive physical therapy for 45 minutes per session 3 times a week for 16 weeks. The therapy protocols will be individualized for every participant according to their level of impairment and preset goals, based on the principles of intensive repetitive task practice programs such as CIMT and HABIT. These consist of stretching of spastic muscles (activity-based dynamic stretching with child's active involvement to the spastic upper limb muscle, particularly muscles which are required for preparatory techniques will be involved in lengthening) and UE weight bearing exercises (ie, UE weight bearing in fundamental or functional position in the form of scapular and upper thoracic rotation and/or push and pull with a vestibular ball while maintaining corrected scapular positioning). A variety of arm and hand activities will be practiced, such as reaching for rings, removing and putting them back, ball throwing, opening a bottle cap, turning a door knob, clay activities, picking marbles from sand, and putting pellets and pegs into sockets, etc.

### Experimental Group Intervention

A typical session for the experimental group (n=70) will consist of stretching exercises followed by the game-based exercise program. Similar to the concept of “shaping,” and consistent with CIMT principles, CGR takes advantage of ergonomic properties of common objects to amplify limited and small amounts of voluntary movement and then allows opportunities for an appropriate switch to objects having more demanding movement requirements or functional demands. CGR allows object properties (size, weight, texture, and surface properties) to be easily manipulated in therapy. This provides graded practice for activities that need to be repeated in daily activities and in play. An important element of the platform is the ability to incorporate movement precision. In this regard, we target finger-hand function and not just transport and/or reaching movements.

Different computer games require different levels of movement amplitude, speeds, precision levels, as well as repetition and appeal to individual preferences. Furthermore, many inexpensive “off the shelf” computer games have a broad range of visuospatial cognitive content. Knowledge of the therapeutic value (object and games activities) can allow the therapist to prescribe an integrated program to target specific goals, for example, speed, accuracy, endurance, visuospatial functions, and cognitive inhibition, and to exercise 2 or 3 fingers, the whole hand, and bimanual tasks tailored to individual child abilities.

**Figure 1:**
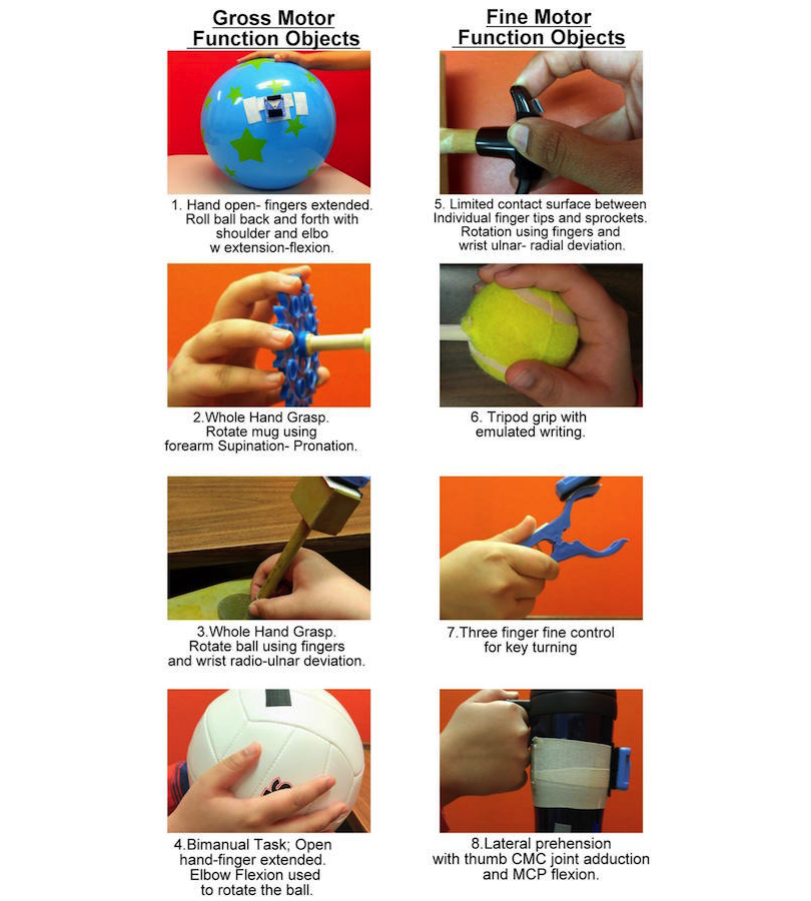
Descriptions of the object manipulation tasks and their respective therapy values for assessment and/or treatment. A miniature, wireless motion mouse is attached with Velcro to each object. Each object manipulation task has specific fine or gross motor skill qualities for therapeutic exercise or ergonomic properties.

In a manner similar to interval training, 6 to 8 objects selected for specific therapy goals will be used to play computer games. Objects can be selected for motor skill training of finger/wrist or elbow and shoulder motions, and also of bimanual controls. Each object manipulation exercise will be used for 2 to 4 minutes. A number of possible objects which, when instrumented with the motion mouse, can be used for the game-based exercise program is shown in Figure 1 (see also Szturm et al [31]). Many action and cognitive-type computer games are available to play; these will be selected by the treating therapist based on (1) degree of difficulty; (2) movement amplitude, speed, and accuracy; (3) visual-spatial processing requirement; and (4) personal preferences of the child. Common computer games that will be used in the game-based exercise program are shown in Textbox 1.

**Computer games that will be used in the exercise program.**

Computer game

1. Aqua Ball and Action Ball.
  - Horizontal, single-axis brick buster with slow to moderate speed, and low to moderate movement precision.
  - Small to moderate number of distracters and simple to complex 2D backgrounds.
2. Jar of marbles and butterfly escape.
  - Horizontal, single-axis matching colors with slow to moderate speed and low to moderate movement precision.
  - Small to moderate number of distracters and simple and moving backgrounds.
3. Owls and bubbles.
  - A single-axis game that requires the player to move the mouse cursor on bubbles to pop them and free the owls to fly away.
4. Hummingbird.
  - A single-axis game that requires the player to move the bird up and down so that it touches the flowers.
5. Feeding frenzy.
  - Two-axis game play with slow motion element and low to moderate movement precision.
  - Moderate to large number of distracters.

### Primary Outcome Measures

The Quality of Upper Extremity Skills Test (QUEST) is a commonly used outcome measure that evaluates quality of dissociated movements, UE gross motor function, and object manipulation in children with CP. It consists of 36 tasks evaluated in 4 domains: dissociated movement, grasp, protective extension, and weight bearing. The tool has demonstrated excellent test-retest reliability [42], and through construct validity studies, has been demonstrated as a good measure of UE motor skill [43].

The following subtests of the Peabody Developmental Motor Scale, Second Edition (PDMS-2) will be used: (1) object manipulation (24-item subtest that measures a child's ability to manipulate balls); (2) grasping (a 26-item subtest that measures a child's ability to use his or her hands [44]); and (3) Visual-Motor Integration (VMI) subtest (a 72-item subtest that measures a child's ability to use visual perceptual skills). Both the PDMS-2 fine motor composite score and the VMI subtest score have shown high test-retest reliability and have good construct validity [45,46].

Immediately following the 16-week intervention, a semi-structured interview format will be used to ask parents about the 5 most important activities their child was trying or wanted to do, but was having difficulty performing and/or difficulty in retaining.

## Results

We will test the difference between the experimental and control groups on the QUEST and PDMS-2 outcome measures using analysis of covariance (ANCOVA); the dependent variable will be the post-intervention measurement of the outcome and the covariates will be the pre-intervention measurement and group membership as the between-subjects effect. Residual diagnostics will be carried out for the ANCOVA model and if their normality assumption fails to hold, appropriate transformations of the response, such as logarithmic, will be explored. Based on published data for the primary outcome measures (QUEST and PDMS-2) [37-41], a power analysis was conducted to determine the required sample size to test the difference between the experimental and control groups using the ANCOVA model. We selected the case (QUEST) which gave the largest sample size. Assuming a correlation of at least 0.6 between baseline and final outcome, then with a sample size of 128 and a standard deviation of 25 we will be adequately powered to detect a difference of 10 units with 80% power, and 5% alpha post intervention. We expect an attrition rate of 10% over the study period. Given this, we propose to recruit a sample of 140 children to participate in equal numbers to be randomized to each group. All calculations were made with PROC GLMPOWER of SAS version 9.3 (SAS Institute, Cary, NC).

Feasibility will be evaluated on the basis of the 2010 Thabane et al model [47] which evaluates 4 domains: process, resources, effectiveness, and human and data management. Process evaluates feasibility of key study processes, such as participant recruitment rates, dropout rates, eligibility criteria, and participant retention rates. Resources, such as time taken to complete study assessments and other resource problems, will be reported over the study period by the project site leads from each site and the data will be compiled.

The parents of the children and the therapist experiences with the interventions and tools will be explored using semi-structured interviews using the qualitative description approach. The following open-ended questions will be asked of the children's parents: (1) when you agreed to participate, how did you hope your child would benefit from the therapy program? (2) Were there things about the game (or exercise therapy program) for your child you liked and things you did not like? (3) What did you think about the computer games your child was asked to play? Did your child enjoy the games? Were there games which your child did not seem to enjoy? (4) Did you feel that this therapy program helped your child? (5) If you were provided with the right setting, would you want your child to continue with these exercises?

The following open-ended questions will be asked of each treating therapist who delivered the game-based therapy program: (1) compared to usual therapy exercises how easy or difficult was it to implement the game exercise program for the children? (2) What kind of difficulties did you face, if any, regarding the use of the motion mouse or other parts of the technology? (3) What qualities did the computer game based intervention possess, if any, that made it more engaging and fun for the children than the conventional protocol? (4) Why would you like to recommend this intervention and technology to your peers, colleagues, and patients? (5) Are there any thoughts, queries, or doubts regarding this treatment method that you would like to express or discuss with us?

The responses of the parents and therapists will be analyzed with content analysis methods using the descriptive as well as interpretative approaches [48,49]. The data collected during the semi-structured interviews will be in the local language. Responses will be transcribed and translated by authorized personnel to organize the data by labelling, structuring, and familiarizing processes. Each participant’s data will be reviewed and analyzed by 2 researchers. Their narrative summary will then be sent back to the parents and therapist for review and approval to ensure trustworthiness of the transcribed and summarized data. Direct quotes from parents’ and physiotherapists’ interviews will be used while writing the descriptive report to illustrate a range of issues faced during the study, behaviors, experiences, and opposing views of participants and strategies. These will be used to develop general statements and hypotheses, which can be tested in subsequent studies. A second order analysis will take place by creating a coding plan based on the research questions. Once the data is coded and sorted, these responses will be categorized to identify themes. The recurrent themes and response clusters are helpful to build event sequences.

## Discussion

Emerging game-based rehabilitation technologies have the potential to improve child participation in repetitive task practice, and therefore, enhance function. The purpose of the study is to provide evidence of the therapeutic value of CGR to improve UE motor function for children with CP. CGR is designed to be used with modern, common computer games, which are low-cost and easily available. Commercial games offer a wide range of levels of precision and movements that vary in speed, amplitude, direction, and accuracy. There is also a wide range of executive cognitive activities available in commercial games for children. It is important to have a large variety of exercise and cognitive activities in games to maintain high levels of motivation and interest among participating children. Knowledge of the therapeutic value (object and games) can allow the therapist to prescribe an integrated program to target specific goals.

The qualitative findings of participants and therapists will help to identify the perceived exercise benefits, any difficulties with the exercises and using the technologies, the engagement and motivational value of the computer games, personal and environmental factors that may have influenced doing the exercises, and any recommendations and modifications for improving the exercise programs.

## Appendix

Multimedia Appendix 1 CONSORT flow diagram.

Multimedia Appendix 2 CONSORT checklist.

## Abbreviations

ANCOVA: analysis of covariance
CIMT: constraint-induced movement therapy
CP: cerebral palsy
GMFCS: Gross Motor Function Classification Scale
HABIT: hand-arm bimanual intensive therapy
MACS: Manual Ability Classification System
PDMS-2: Peabody Developmental Motor Scale, Second Edition
QUEST: Quality of Upper Extremity Skills Test
UE: upper extremities
VMI: Visual-Motor Integration
