# Supplementary material for: The Effectiveness of a Computer Game-Based Rehabilitation Platform for Children With Cerebral Palsy: Protocol for a Randomized Clinical Trial
Source: JMIR Res Protoc. 2017 May 18;6(5):e93. doi: 10.2196/resprot.6846 (PMC5454217; doi:10.2196/resprot.6846)
Supplement: Multimedia Appendix 1 [file resprot_v6i5e93_app1.pdf]

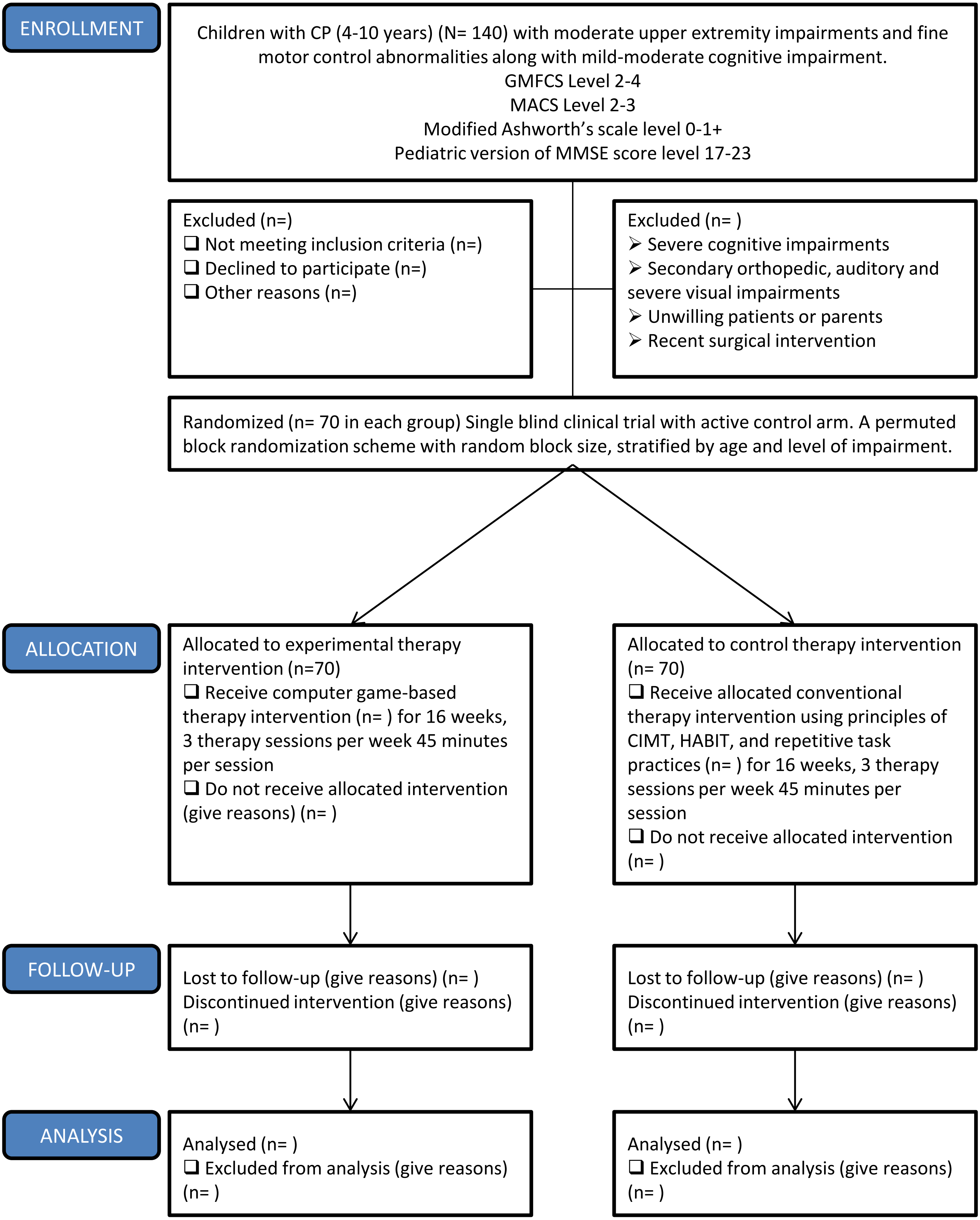
ENROLLMENT
Children with CP (4-10 years) (N= 140) with moderate upper extremity impairments and fine motor control abnormalities along with mild-moderate cognitive impairment.
GMFCS Level 2-4
MACS Level 2-3
Modified Ashworth's scale level 0-1+
Pediatric version of MMSE score level 17-23
Excluded (n=)
❑ Not meeting inclusion criteria (n=)
❑ Declined to participate (n=)
❑ Other reasons (n=)
Excluded (n=)
➢ Severe cognitive impairments
➢ Secondary orthopedic, auditory and severe visual impairments
➢ Unwilling patients or parents
➢ Recent surgical intervention
Randomized (n= 70 in each group) Single blind clinical trial with active control arm. A permuted block randomization scheme with random block size, stratified by age and level of impairment.
ALLOCATION
Allocated to experimental therapy intervention (n=70)
❑ Receive computer game-based therapy intervention (n=) for 16 weeks, 3 therapy sessions per week 45 minutes per session
❑ Do not receive allocated intervention (give reasons) (n=)
Allocated to control therapy intervention (n= 70)
❑ Receive allocated conventional therapy intervention using principles of CIMT, HABIT, and repetitive task practices (n=) for 16 weeks, 3 therapy sessions per week 45 minutes per session
❑ Do not receive allocated intervention (n=)
FOLLOW-UP
Lost to follow-up (give reasons) (n=)
Discontinued intervention (give reasons) (n=)
Lost to follow-up (give reasons) (n=)
Discontinued intervention (give reasons) (n=)
ANALYSIS
Analysed (n=)
❑ Excluded from analysis (give reasons) (n=)
Analysed (n=)
❑ Excluded from analysis (give reasons) (n=)
