# Supplementary material for: The Effectiveness of a Computer Game-Based Rehabilitation Platform for Children With Cerebral Palsy: Protocol for a Randomized Clinical Trial
Source: JMIR Res Protoc. 2017 May 18;6(5):e93. doi: 10.2196/resprot.6846 (PMC5454217; doi:10.2196/resprot.6846)
Supplement: Multimedia Appendix 2 [file resprot_v6i5e93_app2.pdf]

**CONSORT checklist for RCT protocol**

**The Effectiveness of a Computer Game-Based Rehabilitation Platform for Children With Cerebral Palsy: Protocol for a Randomized Clinical Trial.**

| PAPER SECTION And topic | Item | Description | Reported on page # |
|---|---|---|---|
| *TITLE & ABSTRACT* | 1 | How participants were allocated to interventions (e.g., "random allocation", "randomized", or "randomly assigned"). | 3 |
| *INTRODUCTION* Background | 2 | Scientific background and explanation of rationale. | 2 |
| *METHODS* Participants | 3 | Eligibility criteria for participants and the settings and locations where the data were collected. | 3 |
| Interventions | 4 | Precise details of the interventions intended for each group and how and when they were actually administered. | 3-5 |
| Objectives | 5 | Specific objectives and hypotheses. | 2 |
| Outcomes | 6 | Clearly defined primary and secondary outcome measures and, when applicable, any methods used to enhance the quality of measurements (e.g., multiple observations, training of assessors). | 5 |
| Sample size | 7 | How sample size was determined and, when applicable, explanation of any interim analyses and stopping rules. | 5 |
| Randomization -- Sequence generation | 8 | Method used to generate the random allocation sequence, including details of any restriction (e.g., blocking, stratification). | 3 |
| Randomization -- Allocation concealment | 9 | Method used to implement the random allocation sequence (e.g., numbered containers or central telephone), clarifying whether the sequence was concealed until interventions were assigned. | 3 |
| Randomization -- Implementation | 10 | Who generated the allocation sequence, who enrolled participants, and who assigned participants to their groups. | 3 |
| Blinding (masking) | 11 | Whether or not participants, those administering the interventions, and those assessing the outcomes were blinded to group assignment. If done, how the success of blinding was evaluated. | 3 |
| Statistical methods | 12 | Statistical methods used to compare groups for primary outcome(s); Methods for additional analyses, such as subgroup analyses and adjusted analyses. | 5 |
